# Characterization of the Auxin Efflux Transporter PIN Proteins in Pear

**DOI:** 10.3390/plants9030349

**Published:** 2020-03-10

**Authors:** Liying Qi, Ling Chen, Chuansen Wang, Shaoling Zhang, Yingjie Yang, Jianlong Liu, Dingli Li, Jiankun Song, Ran Wang

**Affiliations:** 1 College of Horticulture, Qingdao Key Lab of Genetic Improvement and Breeding of Horticultural Plants, Qingdao Agricultural University, Qingdao 266109, China; qiliying88@163.com (L.Q.); 18254220858@163.com (L.C.); m17864291519@163.com (C.W.); wuhuaguoyyj@163.com (Y.Y.); liujianlong1357@163.com (J.L.); qauldl@163.com (D.L.); qausjk@126.com (J.S.); 2 Institute of Soil and Fertilizer & Resource and Environment, Jiangxi Academy of Agriculture Sciences, Nanchang 330200, China; 3 College of Horticulture, Nanjing Agricultural University, Nanjing 210095, China; slzhang@njau.edu.cn

**Keywords:** *Pyrus*, auxin transport, dwarfing

## Abstract

PIN-FORMED (PIN) encodes a key auxin polar transport family that plays a crucial role in the outward transport of auxin and several growth and development processes, including dwarfing trees. We identified a dwarfing pear rootstock ‘OHF51’ (*Pyrus communis*), which limits the growth vigor of the ‘Xueqing’ (*Pyrus bretschneideri* × *Pyrus pyrifolia*) scion, and isolated 14 putative PbPINs from the pear *Pyrus bretschneideri*. The phylogenic relationships, structure, promoter regions, and expression patterns were analyzed. PbPINs were classified into two main groups based on the protein domain structure and categorized into three major groups using the neighbor-joining algorithm. Promoter analysis demonstrated that *PbPINs* might be closely related to plant growth and development. Through quantitative real-time PCR (qRT-PCR) analysis, we found that the expression patterns of 14 *PbPINs* varied upon exposure to different organs in dwarfing and vigorous stocks, ‘OHF51’ and ‘QN101’ (*Pyrus betulifolia*), indicating that they might play varying roles in different tissues and participated in the regulation of growth vigor. These results provide fundamental insights into the characteristics and evolution of the PINs family, as well as the possible relationship between dwarfing ability and auxin polar transport.

## 1. Introduction

Dwarfing and high plant population density are the inevitable trends of fruit production owing to their important features such as earlier fruiting, higher yields, and ease of management. Currently, application of growth-regulating agents is sometimes used to control tree vegetative growth [1,2,3,4] and many dwarfing rootstocks or interstocks of fruit trees are also widely used to control tree size [5,6,7,8]. Despite its broad utility and the availability of related research on dwarfing rootstocks/interstocks, the underlying mechanism of rootstock/interstock-induced dwarfing is still unknown.

It has been suggested that the dwarfing physiological mechanism might be related to the vessel dimensions, hormone metabolism, and phenol contents of rootstocks [9], as well as the disorder of vascular organization reestablishment and the resultant decline in material transport capacity in the graft union.

In apple trees, it has been reported that the content, transport, and signaling of hormones might play roles in rootstock/interstock-induced dwarfing. For example, the Gibberellic Acid (GA) content in the xylem of dwarfing rootstock ‘M9’ is lower than that of vigorous rootstocks [10,11], and the application of GA to scions on ‘M9’ increased node number [12]. Abscisic Acid (ABA) content is also higher in dwarfing rootstocks [13,14]. Indole Acetic Acid (IAA)content in leaves and branch-barks of scions grafted on the dwarfing interstocks were lower than that of invigorating combinations, but after bridging and substituting interstock bark with invigorating interstock, IAA content in scion gradually restored to a standard tree’s level [15,16]. When auxin transport in the stem was inhibited by polar auxin inhibitor 1-N-naphthylphtha-lamic acid (NPA) in vigorous rootstocks, root zeatin level, and shoot growth declined dramatically [17]. Thus, at present, the best-supported model proposes that dwarfing rootstocks decrease auxin polar transport, thereby reducing root growth and the amount of root-synthesized cytokinin supplied to the scion, which in turn limit the growth of the canopy [9,13]. The auxin polar transport is mainly mediated through auxin carriers, including AUXIN RESISTENT1/LIKE AUX1 (AUX/LAX) influx carrier, PIN-FORMED (PIN) efflux carriers, and ATP binding, cassette B/P-glycoprotein/Multidrug-resistance (ABCB/MDR/PGP) efflux/condition carriers [18,19,20].

The different contents of IAA have been shown to possibly result from the expression levels of the *PIN* gene family members [21]. The PIN-FORMED (PIN) auxin efflux carriers are the major proteins that control polar auxin transport (PAT), which is the unidirectional transporting IAA from the top of the plant morphology to the lower parts [22,23,24,25]. PIN proteins have generally been investigated in *Arabidopsis thaliana*. To date, eight PIN members (AtPIN1 to AtPIN8) have been reported [26] and divided into two categories according to the cell localization. AtPIN1, 2, 3, 4, and 7 are localized on the plasma membrane (PM), mainly responsible for regulating intercellular auxin movement of plants. On the other hand, AtPIN5 and AtPIN8 are localized in the endoplasmic reticulum (ER) [27,28] and have been reported to balance cellular auxin homeostasis. At the same time, PIN proteins have a conserved structure, with trans-membrane helices at the N and C terminals, and some contain an intracellular hydrophilic loop domain [29]. Therefore, based on the presence of the central hydrophilic loop domain, the eight AtPINs can be divided into another two categories. AtPIN1, 2, 3, 4, and 7, with a long hydrophilic loop separating two extremely conserved hydrophobic domains are categorized as canonical PINs. AtPIN5 and AtPIN8 with the absence of the central hydrophilic loop belong to the non-canonical PINs category. AtPIN6 has been reported to be different from other PINs as it is localized on the plasma membrane as well as endoplasmic reticulum [30]. In addition, the hydrophobic loop of PIN6 appeared to have Ser/Thr phosphorylation sites, which were usually a feature of canonical PINs.

PIN proteins have been indicated to play various roles in plant developmental processes [19,31], including embryo development [32,33], lateral root development, shoot vascular development, and floral bud formation, as well as hormone and abiotic stress responses [34,35,36]. For example, *AtPIN1* is mainly involved in the maintenance of embryonic auxin gradients, and organ initiation is severely affected in *pin1* mutants, which results in the formation of naked inflorescence stems [16]. PIN2 has been reported to be the pivotal PIN in regulating root meristem swelling [16]. In rice, the *OsPIN1b* gene has been reported to play roles in auxin-dependent adventitious root emergence and tillering [34]. The transport of auxin from shoot to the root-shoot junction reportedly increased together with tiller number in *OsPIN2* overexpressing plants and resulted in lowered plant height [37]. *OsPIN3t*, is involved in drought stress response and drought tolerance [38]. In addition, *OsPIN9*, *OsPIN10a*, and *OsPIN10b*, are expressed at high levels in adventitious root primordia and pericyclic cells at the stem base, suggesting that they might be involved in adventitious root development. In maize, the expression levels of most *ZmPIN* genes were induced in shoots and reduced in roots by various abiotic stress treatments [34,35]. *ZmPIN1a* and *ZmPIN1b* analysis has also established their involvement in endosperm and embryonic development [39,40]. Eleven *PIN* genes have been identified in sorghum and all *SbPIN* genes were induced by IAA [41].

To date, a series of PIN family members have been reported in some species, including *Solanum lycopersicum* [42], *Glycine max* [43], *Nicotiana tabacum* [44], *Brassica rapa* [45], and *Citrullus lanatus* [15], as well as some perennial woody plants, such as *Vitis vinifera* [25], *Populus trichocarpa* [46], and *Eucalyptus* [47]. Their functions are similar, with some related to root development and most responding to auxins or abiotic stress. However, there is almost no research on *PIN* genes related to dwarfing; there are no genome-wide analyses of *PIN* gene families in *Pyrus*. In this study, we performed a genome-wide analysis of the *PIN* family in pear and analyzed the expression profiles of *PbPINs* in different organs of dwarfing and vigorous rootstocks: ‘QN101’ (*Pyrus betulifolia*) and ‘OHF51’ (*Pyrus communis*). As the scion used in those pear-grafting combinations was ‘Xueqing,’ which came from crosses between ‘Xuehuali’ (*Pyrus bretschneideri)* and ‘Nijisseiki’ (*Pyrus pyrifolia)*, the *Pyrus bretschneideri* genome was selected for PIN-FORMED (PIN) identification. To our knowledge, this is the first study focusing on the identification and expression patterns of *PbPINs*. Our data provides a solid basis for future functional genomic studies on the *PIN* gene family and further research on the dwarfing mechanism. In addition, the identification of *PIN* genes in *Pyrus* will help in understanding the molecular structure and evolution history of *PIN* genes in plants.

## 2. Results

### 2.1. Dwarfing Pear Rootstock ‘OHF51’ Limits the Growth Vigor of the Scion

Fruit quality and tree structural data were collected from two different pear-grafting combinations, ‘Xueqing’/‘QN101’/‘Douli’ and ‘Xueqing’/‘OHF51’/‘Douli.’ As shown in Table 1, when the mean fruit weight was 537.0 g in the combination of ‘OHF51’ used as interstock, which was greater than that in the combination of ‘QN101’ used as interstock (443.2 g). The total soluble solids (TSS) and firmness of fruits between two combinations showed no markedly difference. The tree height was lesser in ‘OHF51’ combination (2.62 m) than that in ‘QN101’ combination (2.93 m) (Table 1). The trunk diameter of scion grafted on ‘OHF51’ was thinner than that grafted on ‘QN101.’ Moreover, the diameter ratios of scion/interstock/rootstock were also different between the two grafting combinations, which were 0.76/1/0.96 in the ‘OHF51’ combination and 0.96/1/1.14 in the ‘QN101’ combination. Those results indicated that ‘OHF51,’ which is a pear dwarfing stock, could limit the growth vigor of the scion and affect the growth of rootstock. However, scion grafted on ‘OHF51’ could lead to increased fruit yield.

### 2.2. Genome-Wide Identification of PIN Genes in Pyrus Bretschneideri

In order to understand if the dwarfing ability of rootstocks was related to the decrease of auxin polar transport, 14 putative *PbPINs* in *Pyrus* were identified. Upon searching SMART (Simple Modular Architecture Research Tool) and Pfam databases using the corresponding protein sequences, the 14 *PbPINs* were all confirmed to encode memtrans-containing proteins, which belong to the *PIN* family. Related information on gene locus IDs, genomic positions, coding region lengths, and the corresponding proteins of *PbPIN* genes are listed in Table 2.

### 2.3. Phylogenetic Relationship Analysis of the PbPIN Family Genes

Several studies have investigated the biological functions of the PIN auxin efflux carriers in *Arabidopsis* [48,49]. Investigation of the evolutionary relationships of PINs from *P. bretschneideri* and *Arabidopsis* will aid in understanding the possible biological functions of these auxin carriers in *Pyrus*.

**Figure 1:**
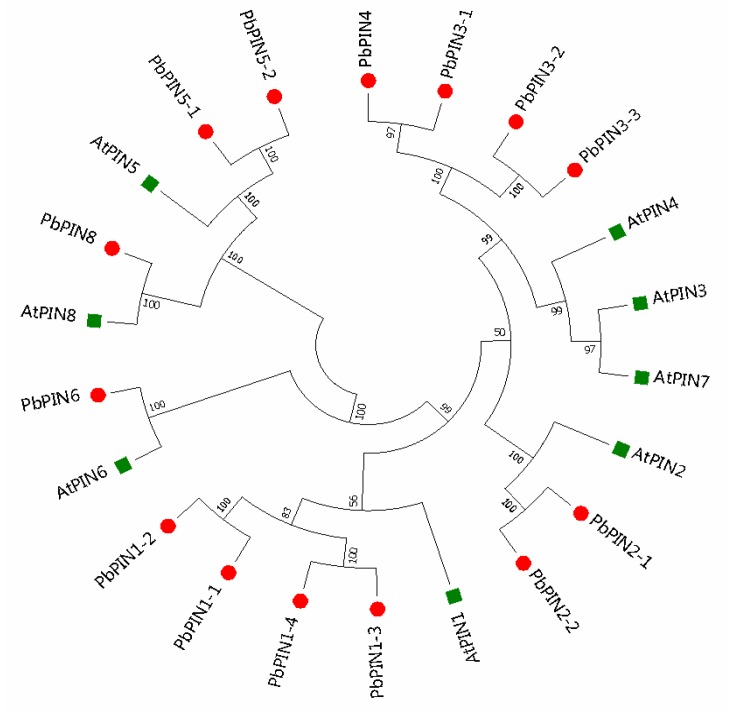
Phylogenetic relationships of PINs between *Pyrus bretschneideri* and *Arabidopsis*. Complete alignments of 22 PINs from *P. bretschneideri* and *Arabidopsis* were performed to construct a phylogenetic tree using MEGA 7.0 software with the neighbor-joining method. Bootstrap values are indicated on the nodes of branches. The green dots and red squares represent AtPINs and PbPINs, respectively. The 14 PbPINs were denominated according to the sequence alignments and the phylogenetic tree.

Phylogenetic analysis of 22 PIN proteins, including eight AtPINs and 14 PbPINs, was carried out using MEAG7.0 software using the neighbor-joining method. The results showed that most PbPINs were clustered with their orthologs in *Arabidopsis* with a high bootstrap support, such as PIN2s, PIN5s, PIN6s, and PIN8s (Figure 1). Some paralogs also existed in pear-like PbPIN1-4/PbPIN1-3, PbPIN2-1/PbPIN2-2, and PbPIN5-1/PbPIN5-2. Adopting the homology of the family members of *Arabidopsis*, the PIN family members of pear were systematically named PbPIN1-1 to PbPIN8, respectively.

### 2.4. Exon-Intron Structure of PbPINs

**Figure 2:**
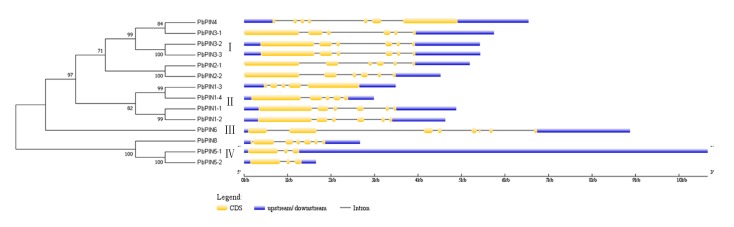
Phylogenetic relationship and exon-intron structure of *PbPINs* in *Pyrus*. The neighbor-joining tree was constructed using MEGA 7.0. Bootstrap values are indicated on the nodes of branches. The yellow boxes denote CDS (Coding Sequence), the blue lines show UTR (Untranslated Regions), and the black lines represent intron.

To investigate the structures of the PIN genes, the numbers and locations of exons and introns were identified using GSDS v2.0 (http://gsds.cbi.pku.edu.cn/) (Figure 2).

Although the number of exons among these *PbPIN* genes is different, changing from three to eight, the genetic structures of most *PbPINs* are highly conserved, and seven out of the 14 *PbPINs* all contained six exons. In addition, these *PbPINs* were categorized into four major groups using the neighbor-joining algorithm. The six genes clustered into Group I exhibited similar exon-intron structures: five members contained six exons, except for *PbPIN4*, which contained seven exons. However, the members of Groups II and IV contained different numbers of exons. In Groups II, *PbPIN1-3* and *PbPIN1-4* had five exons, and *PbPIN1-1* and *PbPIN1-2* had six exons. In Groups IV, *PbPIN5-1* and *PbPIN5-2* both had three exons, and *PbPIN8* contained seven exons. *PbPIN6,* the only one member in group III, had eight exons. The number of introns implied different lengths of these genes. In addition, the sequence lengths of intron or untranslated region were variable, which also affected the length of genes. For example, *PbPIN5-1*, which was approximately 9 Kb long, had a long untranslated region, and *PbPIN6* harbored a relatively longer intron.

### 2.5. Analysis of Conservative Domain of PIN Family

**Figure 3:**
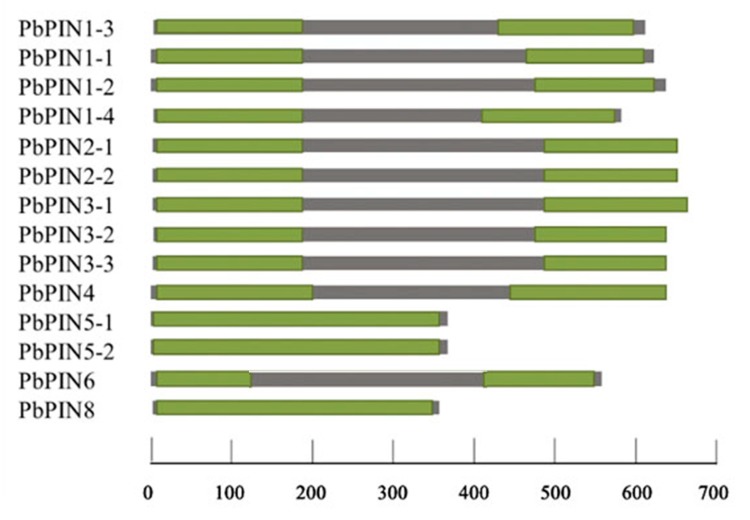
Structures of PbPINs in pear. The name of each corresponding protein is shown on the left. The green color indicates the position of the corresponding conserved domains: transmembrane domains (TMDs). The scale bar represents 100 amino acids.

The length of PIN family members varied widely. We categorized all members into two groups: the long PbPINs (PbPIN1s, PbPIN2s, PbPIN3s, PbPIN4, and PbPIN6), and the short PbPINs (PbPIN5-1, PbPIN5-2, and PbPIN8) (Figure 3).

**Figure 4:**
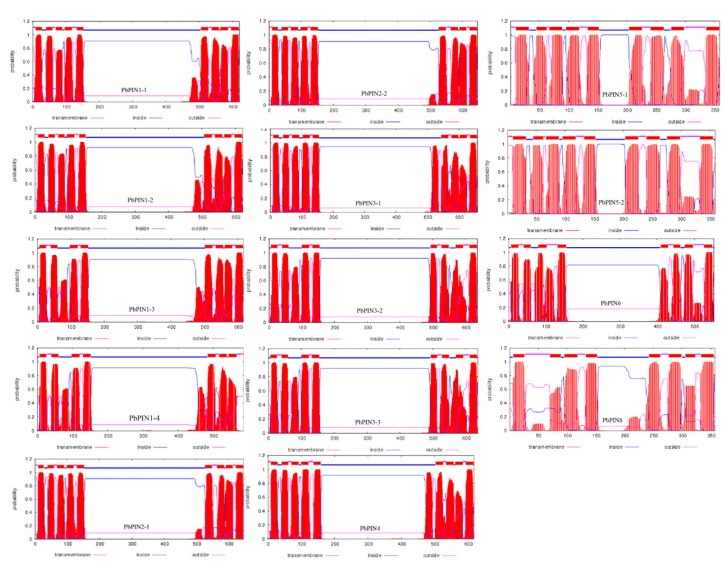
Transmembrane topology analysis of pear PbPIN proteins. The protein transmembrane topology was predicted by using the TMHHM Server v2.0. The predicted transmembrane helices are shown as red peaks.

The different members contained one or two highly conservative transmembrane domains (TMDs), which comprised 200, 350, or 500 amino acids. Meanwhile, similar to other plant PINs, PbPIN proteins have a highly conserved hydrophobicity profile with 7–9 transmembrane helices located at N and C-termini, and linked by a central hydrophilic loop (Figure 4). The length of the central hydrophilic loop is around 300 amino acids for long members of PbPINs and 50 amino acids for short members of PINs (PIN5-1, PIN5-2, and PIN8).

### 2.6. Promoter Analysis of PbPINs

Specific cis-regulatory elements present in the promoter sequences respond to environmental and growth factors and regulate expression of genes. In order to understand the possible response of *PbPIN* genes, the genomic sequences with 2 kb upstream of the translational start site (ATG) of the *PbPIN* genes were obtained from NCBI and analyzed in the Plant CARE database. More than 80 cis-acting elements in the promoter of *PbPINs* were detected, of which, 33 from at least seven promoters were analyzed (Table 3). On the basis of their functions, the identified cis-regulatory elements were classified into five groups. These comprised the core promoter elements, such as CAAT-box and TATA-box, existing in all promoters of *PbPINs*, hormone responsive related elements, such as TCA (salicylic acid related), CGTCA-motif, TGACG-motif (MeJA related), ABRE (abscisic acid related), GARE-motif, P-box, TATC-box (gibberellin related), and ERE (ethylene related), detected in at least eight promoters, stress-related elements (HSE, MBS, TC-rich repeats, and ARE), contained in more than seven promoters, light responsive related elements, such as Box4, G-box, GT1-motif, GAG-motif, Sp1, Box I, and AAGAA-motif, existing in at least seven promoters, and development related elements, such as circadian, Skn-1_motif, and O2-site, detected in almost everyone. The putative functions of cis-elements are listed in Table 3. These results indicated that the role of *PbPINs* might be closely related to plant growth and development, as well as response to endogenesis and exogenesis signals.

### 2.7. Expression of PbPINs in Different Organs

**Figure 5:**
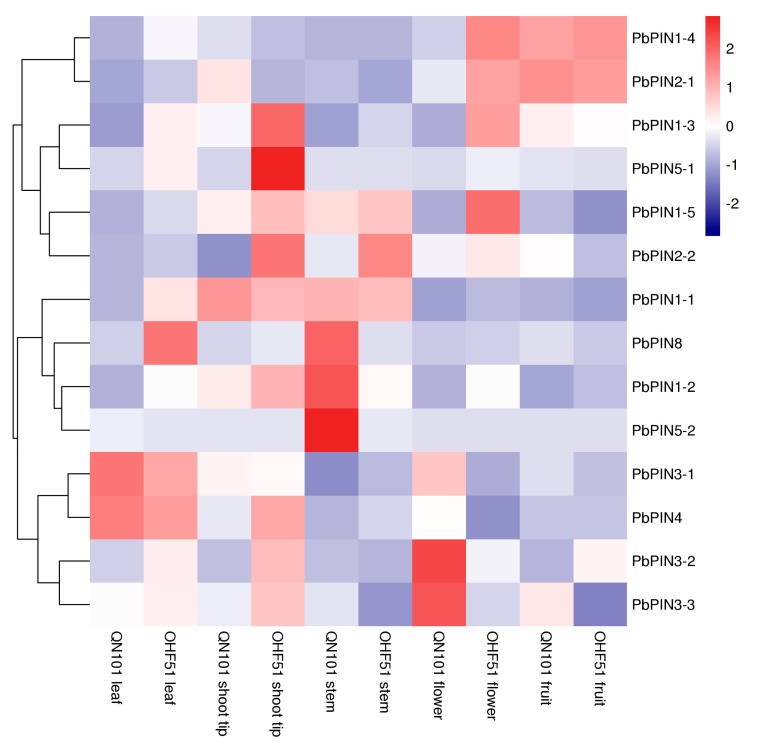
Heat map of the expression level of 14 PbPINs in different organs. The tissues used for the expression profiling are indicated at the bottom of each column. A cluster dendrogram is shown on the left. *PINs* are divided into four major groups based on their expression. The color key at the top right corner represents the Z-score values transformed from log2-based expression values obtained by qRT-PCR.

In order to determine the relationship of *PINs* expression and dwarfing ability of *Pyrus* rootstocks, we detected the expression of *PbPINs* in different organs, including shoot tips, stems, leaves, flowers, and fruits, in vigorous rootstock ‘QN101’ and dwarfing rootstock ‘OHF51’, through qRT-PCR technique. As shown in Figure 5, there was no significantly difference in the expression levels of most *PbPINs* in leaves and fruits between ‘QN101’ and ‘OHF51.’ Only *PbPIN3-1*/*PbPIN4* and *PbPIN1-4*/*PbPIN2-1* displayed higher expression levels in the two tissues. Differences in expression of *PbPINs* between ‘QN101’ and ‘OHF51’ were detected mainly in shoot tips, stems, and flowers. Based on the expression analysis and hierarchical clustering, *PbPINs* were divided into four major groups: Group 1 contained two genes (*PbPIN1-4* and *PbPIN2-1*) with relatively higher expression levels in the fruits of ‘OHF51’ and ‘QN101’ and in the flowers of ‘OHF51.’ Group 2 contained four genes (*PbPIN1-1*, *PbPIN8*, *PbPIN1-2*, and *PbPIN5-2*) whose expression levels were obviously higher in the stem of ‘QN101’ than ‘OHF51,’ and minor differences were detected in leaves and shoot tips between ‘QN101’ and ‘OHF51.’ Group 3 contained four genes (*PbPIN1-3*, *PbPIN2-2, PbPIN5-1*, and *PbPIN6*) with relatively higher expression levels in the shoot tips of ‘OHF51.’ The expression of *PIN2-2* in stems, and *PIN1-3* in flowers was higher in ‘OHF51’ than ‘QN101.’ Group 4 contained four genes (*PbPIN4*, *PbPIN3-1*, *PbPIN3-2*, and *PbPIN3-3*), and relatively higher expression levels were detected in the shoot tip of ‘OHF51’ and the flowers of ‘QN101.’ Higher expression levels of *PbPIN3-1* and *PbPIN4* were also detected in leaves of ‘QN101’ and ‘OHF51.’ These results showed the organ-dependent differences in the expression of *PbPINs.* In the tissues where auxin is transported (shoot tip and stem), the expression levels of *PbPINs* were different in ‘QN101’ and ‘OHF51,’ suggesting that there might be some relationship between dwarfing ability and auxin polar transport in pear stocks.

## 3. Discussion

In commercial apple production, dwarfing rootstocks have been widely used as they can successfully reduce scion vigor, necessitating high-density planting. In pear production, quince (*Cydonia oblonga*) is successfully used as the dwarfing rootstock of *Pyrus communis L*. Furthermore, a series of other *Pyrus* dwarfing stocks have also been reported, such as Old Home × Farmingdale clonal rootstocks [50]. However, the underlying mechanism of rootstock-induced dwarfing remains unclear.

In apple, it has been reported that the contents, transport, and signaling of hormones may play roles in rootstock/interstock-induced dwarfing [9,10,11,12,13]. In leaves and branch-barks of scions grafted on the dwarfing interstocks, IAA content was lower than that of vigorous combinations. However, when the interstock bark was substituted by invigorating interstock, the IAA content gradually restored to a standard tree’s level [15,16]. When auxin transport in vigorous stocks was inhibited by polar auxin inhibitor, zeatin level in root and shoot growth declined dramatically [17]. The results helped to analyze the dwarfing mechanism of rootstocks or interstocks in fruit trees. Thus, at present, the best-supported dwarfing model proposes that lower expression of the *PIN* genes in stem bark tissue leads to the decrease of auxin transport, thereby reduces root growth and synthesis of cytokinin supplied to the scion, and in turn limited the growth of the scion [17,37,51]. However, research on the relationship of *PIN* genes and dwarfing ability in fruit trees are scarce, especially in *Pyrus* plants.

In the present study, we first collected the fruit quality and tree structural data from two different grafting combinations, ‘Xueqing’/‘QN101’/‘Douli’ and ‘Xueqing’/‘OHF51’/‘Douli,’ at 2 years after grafting, which verified the dwarfing ability on the ‘Xueqing’ variety of stock ‘OHF51’ (Table 1). Considering the functions of *PIN* genes in dwarfing ability of apple rootstock and interstock, as well as the importance of pear production, we performed genome-wide comprehensive analysis of the pear *PIN* gene family, based on the white pear genomic data (NCBI; http://www.ncbi.nlm.nih.gov/).

Fourteen members of the PIN family have been found (Figure 1). In *Arabidopsis* [26], *Oryza sativa* [34], and *Populus trichocarpa* [46], as well as almost more than 30 plant species, *PIN* genes have been identified by genome-wide approaches. The lowest number of *PIN* genes is four, which is reported in *Marchantia polymorpha*, in contrast to the highest reported number of *PIN* genes (23) reported in *Glycine max* [43]. It has been suggested that the expanding *PIN* gene family may result from genome duplication. The number of *PIN* genes in pear is around twice of that in *Arabidopsis*. Depending on the phylogenetic analysis, *Arabidopsis* homologous genes widely exist in the pear genome, suggesting that some PIN proteins in *Arabidopsis* and pear may originate from one or more common genes. At the same time, PbPIN1s share low homology with AtPINs in *Arabidopsis*; perhaps merely because of the evolution of individual PIN families in *Arabidopsis* and pear. These phenomena are also found in other species [15,42,44]. PINs were categorized into four major subgroups depending on the neighbor-joining algorithm. Phylogenetic tree analysis with 99 PIN proteins from eight plant species showed that PINs could be divided into seven groups [43]. In *Citrullus lanatus*, phylogenetic tree analysis of 11 ClPIN proteins and eight AtPIN proteins grouped these proteins into five subfamilies [15]. The specificity of these groups in different species may result from the plant evolution.

The exon-intron diversification is also considered to play a role in the evolution of some gene family. Through exon-intron analysis, we found that seven out of 14 *PbPINs* contained six exons, and five of them belonged to subgroup I. This conserved exon-intron organization of *PIN* genes was also found in other plant species [15,31,34,41,46]. Meanwhile, *PbPIN4* in subgroup I contained seven exons; *PbPIN1-3*/*PbPIN1-4* in subgroup II and *PbPIN5-1*/*PbPIN5-2* in subgroup IV contained eight exons and five and three exons, respectively. *PbPIN6* belonging to subgroup III contained eight exons. Although differences existed, members in the same phylogenetic clade, especially the paralogous genes, possessed similar exon-intron structures, such as *PbPIN2-1*/*PbPIN2-2* and *PbPIN3-2*/*PbPIN3-3*, as well as *PbPIN5-1*/*PbPIN5-2*. Therefore, it was speculated that the differences were not an occasional mutation event, but an evolution pattern of plant *PIN* genes. However, *PbPIN4* and *PbPIN3-1* in subgroup I exhibited obviously different exon-intron structure. In addition, we also found two specific members: *PbPIN5-1*, which had a long untranslated region, *PbPIN6*, containing a relatively longer intron. Thus, diversity might still exist in highly conserved genes because of the evolution. At the same time, we compared the exon/intron number between *Arabidopsis* and pear. In *Arabidopsis*, most of the *AtPINs* have six exons and five introns, except for *AtPIN2* and *AtPIN5*, with nine and four exons. Among the orthologous genes between *Arabidopsis* and pear, such as *PIN5s*, *PIN8s*, *PIN2s*, and *PIN6s*, the exon/intron numbers are inconsistent. Additionally, among the paralogous genes in pear, *PbPIN3-2/PbPIN3-3*, *PbPIN2-1/PbPIN2-2*, and *PbPIN5-1*/*PbPIN5-2*, the exon/intron numbers are consistent. It has been regarded that the existence of introns facilitated gene evolution by exon shuffling, allowed the production of multiple proteins from a single gene through alternative splicing, as well as playing a role in gene regulation [52,53]. Thus, we speculate that during the evolution of pear, a series of intron deletion and insertion, gene duplication happened in *PIN* genes, which might cause changes in gene expression and protein function.

In *Arabidopsis* and other plant species, PIN proteins have two highly conserved hydrophobic segments located at N- and C- termini that are linked by a central hydrophilic loop with heterogeneity. Depending on the length divergence of central hydrophilic loop, PIN proteins are grouped into long and short PINs. In *Arabidopsis,* AtPIN1, 2, 3, 4, and 7, which have a long hydrophilic loop between two transmembrane regions, form the canonical PIN proteins, defined as long PIN proteins [29], and are mostly localized to the plasma membrane. The non-canonical PIN proteins (AtPIN5, 6 and 8) have a shorter central hydrophilic loop. They are known as short PIN proteins. AtPIN5 and AtPIN8 are localized to the endoplasmic reticulum (ER), while AtPIN6 has been reported to be localized at the ER and the plasma membrane [30]. In our research, the PbPINs all have two highly conserved hydrophobic segments located at N- and C- terminus, which are linked by a central hydrophilic loop. Similar to other plant PINs, the pear PINs can also be grouped into long PINs, which comprise 11 members (578–666 amino acids in length), and short PINs, which comprise PIN5-1, PIN5-2, and PIN8 (353–377 amino acids in length) according to the predicted protein length (Table 2). In addition, the length of the central hydrophilic loop is around 300 amino acids for members of the long PINs, and 50–100 amino acids for members of the short PINs. In *Arabidopsis*, the long PIN proteins localized in plasma membrane are responsible for the cell-to-cell auxin polar transport [23,24,25], while the majority of the ER-localized short ones are involved in intracellular regulation of auxin homeostasis [24,25,29,48]. Therefore, it can be speculated that the protein lengths are related to the location and function, which may help us to predict the function of orthologous genes of PINs in pear.

Several studies have shown that *PIN* genes are involved in plant growth and development processes. In the present study, the transcript levels of 14 *PbPINs* were analyzed in five different tissues of ‘QN101’ and ‘OHF51,’ the vigorous and dwarfing rootstocks. Our results showed that *PbPINs* had a variety of expression patterns in different organs of different interstocks, mainly in shoot tips, stems, and flowers of ‘OHF51’ or ‘QN101.’ Similar expression patterns have been detected in other species. In rice, *OsPIN5a* and *OsPIN5c* are highly expressed in leaves, shoot apex, and panicle, while *OsPIN5b* is mainly expressed in young panicles [34]. *ZmPIN1b* is highly expressed during female inflorescence development in maize [35]. In watermelon, the *CIPINs* are mainly expressed in mature leaves and stems, and weakly expressed in flowers [15]. More than half of the *NtPIN* genes are highly expressed in stems, *NtPIN5a* and *NtPIN5b* were highly expressed in flowers [44]. The variety of expression patterns in different organs of different interstocks suggested that the *PIN* gene family in pear stock might be related to different functions in growth and development process.

Expression patterns analysis of the 14 *PbPINs* showed that some genes belonging to the same subgroup depending on the neighbor-joining algorithm, such as *PbPIN3-1*, *PbPIN3-2*, *PbPIN3-3*, and *PbPIN4* in subgroup I, had similar expression patterns, suggesting that these genes probably obtained redundant functions during evolution. However, some genes that belong to the same subgroup had divergent expression patterns, such as the paralogous genes, *PbPIN2-1* and *PbPIN2-2,* indicating that these genes obtained unique functions during evolution. In addition, some similar expression of *PbPINs* from different subgroup suggested the functional complementation of *PbPINs*, such as *PbPIN1-4* and *PbPIN2-1*, *PbPIN1-2,* and *PbPIN5-2*.

Previous studies found that dwarfing is due to the lower expression of the *MdPIN1b* gene in stem bark tissue of dwarfing apple interstock, blocking the downward transport of auxin [17]. In *Pyrus communis*, the expression of a *PcPIN-L* is significantly lower in the dwarf-type pears than in standard-type pears. Overexpression of the *PcPIN-L* in tobacco enhances the growth of the stems and the roots [54]. In contrast, overexpression of *OsPIN2* dwarfs the plant height [37]. *ZmPIN1a* overexpression also reduces plant height and internode length [51]. Between ‘QN101’ and ‘OHF51,’ some *PbPINs* displayed different expression patterns. The expression level of *PbPIN8* was higher in leaves of ‘OHF51’ than of ‘QN101.’ In flowers, the expression of *PbPIN3-1*, *PbPIN3-2*, and *PbPIN3-3* was lower in ‘OHF51,’ in contrast to the expression level of *PbPIN1-3, PbPIN1-4,* and *PbPIN2-1*. In addition, a series of genes, such as *PbPIN1-2* in group 2, group 3’s *PbPIN1-3*/*PbPIN2-2*/*PbPIN5-1*/*PbPIN6*, and *PbPIN4*/*PbPIN3-2*/ *PbPIN3-3* in group 4, showed obviously higher expression levels in shoot tips of ‘OHF51’ than that of ‘QN101.’ The expression of *PbPIN8*, *PbPIN1-2,* and *PbPIN5-2* in stem of ‘OHF51’ was also lower than that of ‘QN101.’ It should be noted that *PbPIN2-2* showed a higher expression level in both the shoot tips and stem of ‘OHF51’ than that of ‘QN101.’ We believe that shoot tips and stems are the tissues where auxin is transported. The differences in expression in shoot tips and stems between ‘QN101’ and ‘OHF51’ suggest that the ability of auxin polar transport differs between vigorous and dwarfing stocks, and there may be some relationship between dwarfing ability and auxin polar transport in pear stocks.

## 4. Materials and Methods

### 4.1. Plant Materials and Treatments

The vigorous and dwarfing interstocks, ‘QN101’ (*Pyrus betulifolia*) and ‘OHF51’ (*Pyrus communis*), were grafted on ‘Douli’ (*Pyrus calleryana*) seedlings, respectively, which were cultivated in the pear experimental plot of Qingdao Agricultural University. In the following spring, cultivar ‘Xueqing’ (*Pyrus bretschneideri* × *Pyrus pyrifolia*) was grafted onto the interstocks. Data of tree growth and fruit quality were collected 2 years later. Tree height was recorded after the stop of the annual growth and before pruning. Interstock diameter was evaluated at the middle site; and scion and rootstock diameters were recorded at 5 cm above and below the interstock, respectively. The TSS of the fruit was determined utilizing an ATAGO saccharimeter that had been standardized with distilled water.

‘QN101’ and ‘OHF51’ grafted on ‘Douli’ were used for the expression profiling analysis. Three samples of shoot tips, leaves, flowers, stems, and roots were collected from pear trees grown in the orchard of Qingdao Agriculture University on 15 April 2017, whereas three samples of fruits were collected on 15 October 2017. All samples were immediately frozen in liquid nitrogen and stored at −80 °C until RNA extraction.

### 4.2. Identification and Characteristics of PINs

In order to recognize the auxin efflux carriers (PINs) in Chinese white pear, we searched the corresponding Pear Genome Project (http://peargenome.njau.edu.cn/) [55] and the National Center for Biotechnology Information (NCBI; http://www.ncbi.nlm.nih.gov/) by BLAST (Basic Local Alignment Search Tool) search tools BLASTP and BLASTN [56], using AtPINs sequences as queries which were identified from the TAIR (The Arabidopsis Information Resource) database v10.0 (http://www.arabidopsis.org/). Multiple alignment of the full-length deduced amino acid sequences of PIN proteins was then performed with Clustal X [57], and redundant sequences and different transcripts of the same gene were discarded. SMART (http://smart.embl-heidelberg.de/) [58] and Pfam (http://pfam.san ger.ac.uk/) [59] were used to predict the domain composition of PbPINs. Protein molecular weights (Da) and isoelectric points (pI) were analyzed using the ExPASy proteo- mics server [60]. Alignment of coding sequences with the corresponding genomic DNA sequences was used to determine the CDS-intron structure of PbPINs, and the graphs of exon-intron structures were generated using the Gene Structure Display Server (http://gsds.cbi.pku.edu.cn/) [61].

### 4.3. Sequence Alignment and Phylogenetic Analysis

Multiple sequence alignments of PIN sequences from Chinese white pear (*Pyrus bretschneideri*) and *Arabidopsis*, which were created using ClustalX in MEGA 7.0 with the default parameters [62] were used as the input for the neighbor-joining algorithm in MEGA7.0 to construct phylogenetic trees. Bootstrap analysis was conducted with 1000 replicates to assess the statistical support for each node. Based on the phylogenetic trees, we designated PbPINs following the nomenclature system of the PIN family from *Arabidopsis*.

### 4.4. Sequence Conservation Analysis

Conserved sequences were obtained by SMART and Pfam using the multiple sequence alignment of PbPINs that were created using Clustal X2. The protein trans-membrane topology was predicted using the TMHMM Server v2.0 (http://www.cbs.dtu.dk/services/TMHMM/).

### 4.5. Cis-Acting Regulatory Element Prediction in Promoters Regions of PbPINs

To investigate the putative cis-acting elements in the promoter sequences of *PbPINs*, 2000 bp genomic DNA sequences upstream of the translational start site (ATG) were obtained from the Chinese white pear genome. The upstream sequences were subsequently analyzed and annotated using PlantCARE (http://bioinformatics.psb.ugent.be/webtools/plantcare/html/) [63] for the presence of various cis-acting elements [64].

### 4.6. RNA Extraction and Quantitative Real-Time PCR (qRT-PCR) Analysis

Total RNA was extracted using the RNAprep Pure Plant kit (Tiangen, Beijing, China), following the manufacturer’s instructions. cDNAs were synthesized from moderate total RNA using a PrimeScriptTM RT reagent kit with gDNA Eraser (TaKaRa, Tokyo, Japan). qRT-PCR was performed using the Roche 480 real-time PCR system at the standard mode with the FastStart Essential DNA Green Master kit. All reactions were performed in triplicate at a volume of 20 μL, containing 1 μL of diluted cDNA, and the pear actin gene was used as an internal control. The qRT- PCR amplification was carried out as follows: 95 °C for 5 min, 45 cycles at 95 °C for 15 s, 60 °C for 30 s, and 72 °C for 30 s. *PbPIN* gene-specific primers for quantitative PCR are listed in the Table 4. The relative expression levels of *PbPINs* were calculated using the 2^−ΔΔCT^ method. All qPCR analyses have three biological replicates and two technical replicates.

## Figures and Tables

**Table 1:** Comparison of two types of grafting combinations.

Grafting Combinations	‘Xueqing’/‘QN101’/‘Douli’	‘Xueqing’/‘OHF51’/‘Douli’
Fruit weight (g)	443.2 + 16.0 b	537.0 ± 29.6 a
Total Soluble Solids (TSS) content	13.73 ± 0.16 a	13.56 ± 0.16 a
Firmness (Kg cm^−2^)	6.53 ± 0.12 a	6.42 ± 0.16 a
Tree height (m)	2.93 ± 0.08 a	2.62 ± 0.08 b
Scion diameter (mm)	42.77 ± 2.62 a	33.54 ± 0.98 b
Interstock diameter (mm)	44.63 ± 2.12 a	44.10 ± 1.41 a
Rootstock diameter (mm)	50.78 ± 2.88 a	43.46 ± 2.50 a
Diameter ratio of trunk amongScion/Interstock/Rootstock	0.96/1/1.14	0.76/1/0.96

Note: Data are presented by mean ± SEM (Standard Error of Mean). Means followed by different letters are significantly different at the *P* < 0.05 level using the Students’ *t*-test.

**Table 2:** Characteristics of PIN members in pear.

Gene Name	Gene Locus ID	Genomic Position	CDS Length	Protein Length	Molecular Weight (Da)	Isoelectric Point (PI)	Instability Index	Aliphatic Index
PbPIN1-1	LOC103946937	824,136..828,039	1863	620	67,663.97	9.11	34.52	88.55
PbPIN1-2	LOC103950573	343,331..347,039	1869	622	67,713.01	8.98	34.59	88.62
PbPIN1-3	LOC103933990	216,016..218,808	1854	617	66,514.59	8.89	37.38	94.05
PbPIN1-4	LOC103960490	472,382..474,775	1761	586	63,204.43	9.11	40.01	88.72
PbPIN2-1	LOC103941631	34496..38649	1944	647	69,852.83	9.32	42.75	89.68
PbPIN2-2	LOC103950477	728,493..732,110	1944	647	69,832.76	9.25	43.06	89.98
PbPIN3-1	LOC103947028	312,759..317,361	1980	659	71,419.31	8.47	36.62	90.17
PbPIN3-2	LOC103948593	3,901,582..3,905,923	1917	638	69,255.79	8.2	33.77	92.66
PbPIN3-3	LOC103948670	4,098,128..4,102,477	1917	638	69,255.79	8.2	33.77	92.66
PbPIN4	LOC103931858	51256..56492	1857	618	68,224.38	9.54	38.8	93.12
PbPIN5-1	LOC103930394	305,314..313,840	1080	359	39,296.44	7.65	35.98	116.71
PbPIN5-2	LOC103938552	149,838..151,159	1080	359	39,489.7	7.61	35.38	115.35
PbPIN6	LOC103951142	485,530..492,630	1662	553	60,371.48	8.93	39.93	98.43
PbPIN8	LOC103934837	52,931..55,068	1074	357	38,888.24	9.29	30.69	120.25

**Table 3:** *Cis*-elements present in more than seven promoters of PIN genes in pear.

Cis-Elements	Number of Gene	Functions of the Cis-Elements	Cis-Elements Types
AE-box	8	part of a module for light response	Light responsive
Box 4	12	part of a conserved DNA module involved in light responsiveness	Light responsive
G-Box	11	cis-acting regulatory element involved in light responsiveness	Light responsive
G-box	10	cis-acting regulatory element involved in light responsiveness	Light responsive
GA-motif	7	part of a light responsive element	Light responsive
GT1-motif	11	light responsive element	Light responsive
TCT-motif	8	part of a light responsive element	Light responsive
ATCT-motif	9	part of a conserved DNA module involved in light responsiveness	Light responsive
GAG-motif	13	part of a light responsive element	Light responsive
Sp1	11	light responsive element	Light responsive
Box I	12	light responsive element	Light responsive
CATT-motif	8	part of a light responsive element	Light responsive
I-box	7	part of a light responsive element	Light responsive
AAGAA-motif	11		Light responsive
ABRE	7	cis-acting element involved in the abscisic acid responsiveness	Hormone responsive
CGTCA-motif	11	cis-acting regulatory element involved in the MeJA-responsiveness	Hormone responsive
TGACG-motif	11	cis-acting regulatory element involved in the MeJA-responsiveness	Hormone responsive
TCA-element	11	cis-acting element involved in salicylic acid responsiveness	Hormone responsive
GARE-motif	7	gibberellin-responsive element	Hormone responsive
P-box	7	gibberellin-responsive element	Hormone responsive
ERE	7	ethylene-responsive element	Hormone responsive
HSE	11	cis-acting element involved in heat stress responsiveness	Stress responsive
MBS	9	MYB binding site involved in drought-inducibility	Stress responsive
TC-rich repeats	14	cis-acting element involved in defense and stress responsiveness	Stress responsive
ARE	8	cis-acting regulatory element essential for the anaerobic induction	Stress responsive
Box-W1	8	fungal elicitor responsive element	Stress responsive
W box	8	wounding and pathogen response	Stress responsive
circadian	13	cis-acting regulatory element involved in circadian control	development related elements
Skn-1_motif	13	cis-acting regulatory element required for endosperm expression	development related elements
O2-site	7	cis-acting regulatory element involved in zein metabolism regulation	development related elements
CAAT-box	14	common cis-acting element in promoter and enhancer regions	promoter related elements
TATA-box	14	core promoter element around −30 of transcription start	promoter related elements
Unnamed__1	8	60K protein binding site	site-binding related elements

Note: More than 80 cis-elements in the promoter of 14 PbPINs were detected. We displayed 33 cis-elements, which were detected in at least seven promoters.

**Table 4:** The primer of the RT-PCR reaction.

Name	Sequence	Name	Sequence
*Actin*	U: CCCAGAAGTGCTCTTCCAAC	*PbPIN3-2*	U: ATTTTACCCCTCCCCTCTTCT
	D: TTGATCTTCATGCTGCTTGG		D: AATATCATCGCCACGTACAGC
*PbPIN1-1*	U: ATTGTTCCCTTTGTCTTTGC	*PbPIN3-3*	U: GCCATTCTTAGCACTGCGGTTAT
	D: TTCTCATCATGTTTGCGTTT		D: GGCGGATTCTTCGTTGATCTTCAT
*PbPIN1-2*	U: CCCAGAAACAGAGCAATAAGA	*PbPIN4*	U: CATTCTTAGCACTGCGGTTATT
	D: TGGAGATGAAGTGGAAGGAGA		D: TCGTTCTCCATCGTACTTAATCTA
*PbPIN1-3*	U: GATTGTTCCGTTTGTCTT	*PbPIN5-1*	U: TCTGGGTAGTCCTCAAGTCC
	D: ATATTTCCTCCACGTCTC		D: GTCAGCCGCTATGAACCT
*PbPIN1-4*	U: TTGATTTTCCTTCTGATTTCGG	*PbPIN5-2*	U: ATTAATTACCAGTTGTGCTCCAA
	D: GAAGATTTTCCACCATTTCACG		D: ATCAACATGTGCAGTGAATTCGA
*PbPIN2-1*	U: GCCTTGCCTGTAACAATACT	*PbPIN6*	U: GTTTCCTTACCAGTGACGCTCCT
	D: ATCAAGGTACTCAGTTGTCACAT		D: CATGTACTAATATGACACCAGACCC
*PbPIN2-2*	U: ACGCGGACATACTTAGCACTG	*PbPIN8*	U: CATCCAGACATTTTGAGCAC
	D: TGAACACTGAATCCAATGGCA		D: GTAAACATATCAGCCATATTACA
*PbPIN3-1*	U: CTATTGTCCAGGCTGCTCTTC		
	D: TTCTTCGTTTTCCTAGCTTTGT		
